# In Vitro Biofilm-Mediated Biodegradation of Pesticides and Dye-Contaminated Effluents Using Bacterial Biofilms

**DOI:** 10.3390/microorganisms11092163

**Published:** 2023-08-26

**Authors:** Iram Liaqat, Awais Khalid, Saima Rubab, Farzana Rashid, Asma Abdul Latif, Sajida Naseem, Asia Bibi, Bushra Nisar Khan, Waiza Ansar, Arshad Javed, Muhammad Afzaal, Muhammad Summer, Samia Majid, Sikander Ali, Muhammad Nauman Aftab

**Affiliations:** 1 Microbiology Laboratory, Department of Zoology, Government College University, Lahore 54000, Pakistan; aishansar3@gmail.com (W.A.); muhammad.summer@gcu.edu.pk (M.S.); curator.zoology@gcu.edu.pk (S.M.); 2 Department of Physics, Hazara University, Mansehra 21300, Pakistan; awais.phy@hu.edu.pk; 3 Department of Pharmacognosy, Lahore Pharmacy College, Lahore Medical & Dental College, Lahore 53400, Pakistan; saima.rubab@lmdc.edu.pk; 4 Department of Zoology, Lahore College for Women University, Lahore 54000, Pakistan; dr.farzanarashid@gmail.com (F.R.); asma5latif@gmail.com (A.A.L.); 5 Department of Zoology, University of Education, Lower Mall Campus, Lahore 54000, Pakistan; 6 Department of Zoology, The Women University, Multan 66000, Pakistan; drasia@wum.edu.pk; 7 Institute of Zoology, University of the Punjab, Lahore 54590, Pakistan; 8 Department of Wildlife and Ecology, University of Veterinary and Animal Sciences, Pattoki 55300, Pakistan; arshadjavid@uvas.edu.pk; 9 Sustainable Development Study Centre, Government College University, Lahore 54000, Pakistan; dr.afzaal@gcu.edu.pk; 10 Institute of Industrial Biotechnology, Government College University, Lahore 54000, Pakistandr.naumanaftab@gcu.edu.pk (M.N.A.)

**Keywords:** biofilm, biodegradation, cypermethrin, imidacloprid, Congo red, malachite green

## Abstract

Overuse of pesticides in agricultural soil and dye-polluted effluents severely contaminates the environment and is toxic to animals and humans making their removal from the environment essential. The present study aimed to assess the biodegradation of pesticides (cypermethrin (CYP) and imidacloprid (IMI)), and dyes (malachite green (MG) and Congo red (CR)) using biofilms of bacteria isolated from pesticide-contaminated soil and dye effluents. Biofilms of indigenous bacteria, i.e., *Bacillus thuringiensis* 2A (OP554568), *Enterobacter hormaechei* 4A (OP723332), *Bacillus* sp. 5A (OP586601), and *Bacillus cereus* 6B (OP586602) individually and in mixed culture were tested against CYP and IMI. Biofilms of indigenous bacteria i.e., *Lysinibacillus sphaericus* AF1 (OP589134), *Bacillus* sp. CF3 (OP589135) and *Bacillus* sp. DF4 (OP589136) individually and in mixed culture were tested for their ability to degrade dyes. The biofilm of a mixed culture of *B. thuringiensis* + *Bacillus* sp. (P7) showed 46.2% degradation of CYP compared to the biofilm of a mixed culture of *B. thuringiensis* + *E. hormaechei* + *Bacillus* sp. + *B. cereus* (P11), which showed significantly high degradation (70.0%) of IMI. Regarding dye biodegradation, a mixed culture biofilm of *Bacillus* sp. + *Bacillus* sp. (D6) showed 86.76% degradation of MG, which was significantly high compared to a mixed culture biofilm of *L. sphaericus* + *Bacillus* sp. (D4) that degraded only 30.78% of CR. UV–VIS spectroscopy revealed major peaks at 224 nm, 263 nm, 581 nm and 436 nm for CYP, IMI, MG and CR, respectively, which completely disappeared after treatment with bacterial biofilms. Fourier transform infrared (FTIR) analysis showed the appearance of new peaks in degraded metabolites and disappearance of a peak in the control spectrum after biofilm treatment. Thin layer chromatography (TLC) analysis also confirmed the degradation of CYP, IMI, MG and CR into several metabolites compared to the control. The present study demonstrates the biodegradation potential of biofilm-forming bacteria isolated from pesticide-polluted soil and dye effluents against pesticides and dyes. This is the first report demonstrating biofilm-mediated bio-degradation of CYP, IMI, MG and CR utilizing soil and effluent bacterial flora from Multan and Sheikhupura, Punjab, Pakistan.

## 1. Introduction

Pakistan is one of the developing world’s emerging nations. Its textile industry is the country’s largest manufacturing sector and the world’s fourth largest cotton producer [1]. However, the textile industry of Pakistan is highly polluting with detrimental impacts on the environment and animal health [2]. It demands an extensive supply of water for dyeing, bleaching and printing, which makes it one of the most major wastewater sources that harm the environment [3]. Overuse of pesticides in agriculture results in severe soil and water contamination; for instance, cotton-planted soil is severely contaminated by pesticides because of the continual use of pesticides to obtain maximum output [4].

Cotton is grown extensively in Pakistan’s Multan district and approximately 80% of total pesticides are now applied to cotton plants [5,6]. The soil quality in the Multan district has deteriorated as a result of the excessive use of harmful pesticides for the large-scale production of cotton and wheat. In this study, two pesticides, cypermethrin and imidacloprid, are targeted because they are frequently used on cotton crops in Multan. Cypermethrin [7] (CYP: C_22_H_19_C_l2_NO_3_) is a synthetic pyrethroid pesticide used to control pests in cotton fields, and on fruits and vegetable crops [8]. About 90% of fabricated cypermethrin is preferably applied to cotton crops [7]. It adversely affects the central nervous system and causes allergic skin reactions, such as eye irritation [9]. The half-life of cypermethrin is 30–60 days in soil and water. During this time, it may enter into organisms residing in polluted sites via the food chain and spread to nearby land as well as water resources [10]. Imidacloprid (IMI; C_9_H_10_ClN5O_2_) [11] is a commonly used pesticide in Pakistan, known for its high persistence in soils for a year or more. It belongs to a relatively new class of insecticide, known as neonicotinoids [12]. IMI is sprayed on seeds to control sucking insects in cotton and wheat crops, as well as thrips, whiteflies and jassid in cotton crops [13]. IMI has attracted attention as an efficient pesticide because it is routinely sprayed over fields to protect crops, including rice, cotton, potato, apple, maize and wheat, etc. [14]. It has the potential to move into surface water and leach into groundwater due to high soil mobility [12]. Following application, pesticides are stable initially on leaflets for a period of time, and then drop down as waste foliage into the soil. There may be some bacterial species that may survive on the pesticide-exposed surface of leaves and develop resistance against these as a result of repeated applications.

Due to the unsuitability of the groundwater in Pakistan, textile effluent is often used for the irrigation of farmlands adjacent to major cities [15]. Dyes, including Malachite green (MG), a triphenylmethane dye [16], are widely used to dye wool, cotton, silk and ceramics, reflecting MG’s extensive industrial applications due to its low cost, ready availability and efficiency. These substances are officially declared carcinogenic by the US Food and Drug Administration [17]. They can induce hepatic tumors in rodents and result in aberrant rabbit and fish reproduction. These factors make it crucial to eliminate MG dyes from contaminated water sources [18]. Similarly, Congo red (CR) is an azo dye extensively used in the printing, textile, biomedical and food industries. It can have carcinogenic effects on humans, plants and animals [19]. Azo dyes are highly colored, readily apparent and create significant environmental problems by affecting water transparency as well as creating aesthetic problems. Some of the dyes, their precursors, or their biotransformation products, such as aromatic amines, have been shown to be carcinogenic [20]. As a result, the detoxification of textile effluents is a “hot topic” among environmentalists, government organizations, and researchers all over the world.

Owing to the toxicity concerns, removing such pollutants from the environment in an eco-friendly manner is a significant need to save the environment. There are numerous physical (e.g., adsorption, flocculation, coagulation, membrane filtration, etc.), and chemical (e.g., oxidation process, Fenton’s reagent, ozonation, etc.) techniques available [21]. However, these methods have their own drawbacks, such as low efficiency, sludge production, the production of harmful by-products, and often high costs. Bio-degradation with microorganisms is a less expensive, more viable and environmentally friendly alternative to physiochemical techniques [22].

The potential of microbes to biodegrade dyes or pesticides mainly depends on their metabolic activity. Bacteria are the most important candidates for the degradation of xenobiotics due to their capabilities of fast adaptation and simple organization [23]. Bacterial biofilms are organized groups of bacterial cells that are firmly attached to biotic or abiotic surfaces and are encircled by extracellular polymeric substances (EPS). However, there have been few studies that have examined the role of biofilms in dye and pesticide degradation. There are various advantages of bacterial biofilm over planktonic organisms. The protection of bacteria from harmful stressors (such as toxic chemicals, temperature, pH, and predators), the exchange of genetic material, provision of nutrition, persistence under diverse metabolic settings, and expression of signal molecules are only a few of the factors that make bacteria resistant [24,25]. Microbes in biofilms are able to oxidize harmful organic pollutants in an efficient, cost-effective and environmentally friendly manner when contaminated water passes through a biofilter. Microbial biofilms are more efficient regarding biodegradation as they can absorb, degrade and immobilize many environmental pollutants. Hence, bacterial biofilms are frequently employed in wastewater treatment approaches [26].

Several bacteria, including *Bacillus alkalinitrilicus* [27], *Klebsiella pneumoniae* BCH1 [11], *Pseudomonas* sp. RPT 52 [28], *Stenotrophomonas maltophilia*, *B. thuringiensis*, *Ensifer meliloti*, *P*. *stutzeri*, *Variovorax boronicumulans*, *Fusarium* sp. [29], *Agrobacterium* sp. InxBP2 [30], *Sphingobacterium* sp. InxBP1 [31], *Pseudoxanthomonas*, *Mycobacterium*, *Rhizobium*, *Rhodococcus*, and *Stenotrophomonas*, have been reported to have biodegradation potential for IMI [14]. CYP degradation by *Bacillus* sp. isolated from pesticide-polluted soil of a rice field was previously reported [32]. Similarly, various microorganisms have been identified for the potential degradation of MG, such as *Micrococcus*, *Saccharomyces cerevisiae* and *Pseudomonas* sp. [33]. The *B. cohnni* strain RKS9 was reported to remove 99% of CR dye [34]. Biofilm-forming bacteria indigenous to pesticides and dyes in polluted sites become resistant to the pesticides and/dyes and develop efficient mechanisms for their biodegradation by using them as metabolites. Accordingly, soil collected at contaminated sites provides a good source of microorganisms having the ability to degrade pollutants; thus, these can be used to obtain enriched and selected pesticide-degrading microbial communities.

To the best of our knowledge, this is the first report demonstrating the biofilm-mediated bio-degradation of pesticides (CYP and IMI) and dyes (MG and CR) utilizing soil and dye effluent bacterial flora individually and in mixed culture from Multan and Sheikhupura, Punjab, Pakistan. Samples after biofilm treatment were analyzed through UV–VIS spectroscopy, FTIR and TLC to determine their biodegradation potential.

## 2. Materials and Methods

### 2.1. Chemicals and Pollutants

Commercial grade CYP (EW: 25% cypermethrin) and IMI (WP: 25% imidacloprid) were procured from the agrochemical shop in the Kasur district, Punjab. Analytical grade MG and CR were purchased from Sigma Aldrich, Lahore, Punjab, Pakistan. The stock solutions of the prescribed pesticides and dyes (100 mgL^−1^) were prepared by dissolving in double distilled water, sterilized with a 0.2-micrometre syringe filter, followed by storage at 4 °C until use. All other reagents and chemicals used in this study were of analytical grade.

### 2.2. Soil Sampling and Isolation of Bacterial Strains

IMI and CYP were selected, owing to their popularity for use in farmers’ fields, from the Multan district for the collection of soil samples. Six soil samples for pesticide degradation were obtained from pesticide-polluted cotton crop fields in Shujaabad, Multan, Pakistan (geographical coordinates: latitude 30.12° N, and longitude 71.42° E). The fields had been sprayed by CYP and IMI over the past 7 years. Six dye-contaminated wastewater samples were collected from effluents of the textile industry at Sheikhupura, Lahore, Pakistan (geographical coordinates: latitude 31.62° N, and longitude 74.21° E). The samples were collected in sterile polyethylene bags using random sampling methods from the superficial layer of soil (15–20 cm depth) and brought aseptically to the Microbiology Laboratory, Department of Zoology, Government College, University of Lahore, for further use. The soil and effluent samples were serially diluted into a saline solution (NaCl, 0.85 g) and plated onto nutrient agar. Plates were incubated at 37 °C for 24 h following Firoozeh et al. [35]. After incubation, 16 morphologically distinct colonies, ten from cotton crop soil (termed 2A, 2B, 3A, 3B, 4A, 4B, 5A, 5B, 6A and 6B) and six from dye-polluted effluents (AF1, BF2, CF3, DF4, EF5 and FF6) were purified via quadrant streaking.

### 2.3. Congo Red Assay (Qualitative Biofilm Production Assay)

Congo red agar is composed of a brain–heart infusion (BHI) broth (37 gL^−1^) supplemented with sucrose (50 gL^−1^), Congo red (0.8 gL^−1^) and agar (10 gL^−1^). The CR stain was prepared as a concentrated solution and autoclaved at 121 °C for 15 min independently from the other medium ingredients. It was poured into autoclaved BHI broth with sucrose. All 16 isolates were streaked on CRA plates and incubated at 37 °C for 24 h. Biofilm-forming bacteria appeared black, while non-biofilm-formers showed red color [36,37]. The test was performed in triplicate. Biofilm-forming isolates were selected for further study.

### 2.4. Morphological and Biochemical Characterization

Biofilm-forming isolates were characterized by Gram staining and colony morphology (shape, size, elevation, margin, surface, opacity and color, etc.) under a microscope at 4× power lens. Biochemical tests including catalase, urease, starch hydrolysis, carbohydrate fermentation (glucose, lactose and sucrose) and application of Methyl Red Voges Proskauer (MRVP) broth were performed following Bergey’s manual of systematic bacteriology [38].

### 2.5. Genetic Identification of Bacterial Strains

The strains were identified by 16S rRNA gene sequence amplification following the method of Liaqat and Sabri [39]. In brief, genomic DNA was extracted with the Gene JET Genomic DNA Purification Kit (ThermoFisher Scientific, Waltham, MA, USA). PCR was performed using universal primers PA as forward primers (5′-AGAGTTGATCCTGGCTCAG-3′) and PH as reverse primers (5′-AAGGAGGTGATCCAGCCGCA-3′) in a thermal cycler. A polymerase chain reaction was performed in 25 µL volume, comprising of 1.0 µL genomic DNA (50 ng), 0.2 µL of Taq DNA polymerase (1 U/µL) and 15.9 µL MilliQ grade water. PCR amplification was performed with the following cyclic profile (initial denaturation for 4 min at 95 °C, followed by 30 cycles of denaturation for 30 s at 94 °C, annealing for 40 s at 58 °C and elongation at 72 °C for 1 min, with a final extension at 72 °C for 10 min). The PCR product was checked using 0.8% agarose gel electrophoresis under a UV illuminator. The product was purified with a Purelink^TM^quick Gel Extension Kit (Ref K210012, Invitrogen, Waltham, MA, USA). Samples were sent for sequencing to Axil Scientific, Singapore. Obtained sequences were compared using a similarity index with the type strains from genomic database banks using the NCBI Blast available at https://blast.ncbi.nlm.nih.gov/Blast.cgi?PROGRAM=blastn&PAGE_TYPE=BlastSearch&LINK_LOC=blasthome (accessed on 11 March 2022) and the isolates were taxonomically identified up to species level.

The sequence was submitted to GenBank at https://submit.ncbi.nlm.nih.gov/subs/genbank/ (accessed on 11 March 2022) to obtain accession numbers. A phylogenetic tree was constructed using MEGA X software version 11 by the neighbor-joining method.

### 2.6. Biofilm Time Kinetics

To determine the biofilm time kinetics of the isolated strains, a test tube crystal violet (CV) staining method was used [40]. In brief, 30 μL inocula of bacterial strains already adjusted to 0.5 McFarland turbidity standard were added to 3 mL LB broth in labeled test tubes, followed by incubation at 37 °C for 3, 5 and 7 days. The first set of test tubes was taken out after a three day incubation period. Medium containing bacterial culture was discarded, and test tubes were washed with 0.85% saline solution to remove planktonic cells and air-dried for 20 min. Then, 3 mL of 0.1% crystal violet was added to each test tube. An amount of 33% glacial acetic acid was added and OD was measured at 560 nm using a spectrophotometer. The process was repeated with the remaining two sets of test tubes and examined after 5 and 7 days. The experiment was carried out in triplicates.

### 2.7. Biofilm-Mediated Biodegradation Assay

Biodegradation experiments of pesticides and dyes were performed using bacterial biofilms individually and in mixed culture (Table S1) following slight modifications [17]. Briefly, 30 µL pre-inocula of individual and each of the mixed biofilm consortia (turbidity adjusted to 0.5 McFarland standard) were added in 3 mL nutrient broth in glass test tubes, followed by incubation at 37 °C for 5 days. Afterwards, the broth culture was discarded, and test tubes were washed using 0.85% saline solution to remove planktonic cells. Test tubes containing bacterial biofilms were then supplemented with 3 mL (100 μgmL^−1^ conc.) solution of pesticides (CYP and IMI) and dyes (MG and CR) at pH 7 and incubated for 24 h at 37 °C to determine the degradation ability of the biofilm [11]. Negative controls with only the test pesticides and dyes were incubated under the same conditions. After the incubation period, 1.5 mL aliquots from each culture were centrifuged at 10,000 rpm for 5 min. The pellet containing biofilm was discarded and the supernatants were saved into new tubes. The supernatant (of the samples and controls) was subjected to analysis using a UV–VIS spectrophotometer (AE-S70-1U), London, UK [41]. The degradation of pesticides (CYP and IMI) and dyes (MG and CR) was estimated by measuring the change in absorbance at the maximum absorption wavelength (λ max) of the cultured supernatants.

Degradation was expressed as a percentage (%) using the data obtained from UV–VIS analysis. The following equation was used to calculate the degradation % of the pesticides and dyes.

(1) $$\mathrm{Degradation}\;\mathrm{Percentage}\;(\%)=\left[\frac{\left(A-B\right)}{A}\right]\times 100$$

where A = absorbance of the sample before biofilm treatment and B = absorbance of the sample after biofilm treatment. CYP, IMI, MG and CR degradation were investigated for each individual biofilm and mixed culture bacterial biofilm.

### 2.8. Fourier Transform Infrared Spectroscopy

The supernatant of the treated samples and control was carefully collected and biodegraded metabolites/residues in the supernatant were subjected to FTIR analysis [42]. FTIR of the samples containing the biodegraded CYP, IMI, MG and CR was carried out in the IR range of 500–4500 cm^−1^ to identify the stretching of bonds in the test pesticides and dyes after biodegradation [9]. FTIR analysis was performed only for those isolates that showed maximum biodegradation potential.

### 2.9. Thin-Layer Chromatography(TLC)

Biodegraded products of the selected pesticides and dyes were extracted with ethyl acetate and analyzed by TLC on silica gel following slight modifications according to Kalyani et al. [43]. The solvents used for TLC were: n-hexane, acetone, ethyl acetate (80:10:10) for CYP, toluene and methanol (9:1) for IMI, ethyl alcohol, water, methanol, and amyl alcohol (4:3:2:1) for MG, and water, ethanol, and acetone (4:4:1) for CR. The metabolites were visualized or detected by exposure to iodine vapors. TLC analysis was performed only for those isolates that showed maximum biodegradation potential.

### 2.10. Statistical Analysis

All the experiments were performed in triplicates and the data obtained were expressed as mean ± standard error. One-way analysis of variance (ANOVA) was applied to determine the significant differences between the samples at the 95% confidence level. The data were analyzed using SPSS software version 23 and graphs were constructed using Graph Pad Prism (version 8.0.1) software.

## 3. Results

### 3.1. Screening of Biofilm Producers

The results regarding the screening of biofilm producers are presented in Table S2. Out of 10 bacterial strains isolated from pesticide-contaminated soil samples, 4 (2A, 4A, 5A and 6B) showed black growth on CR agar, hence showing their biofilm-forming potential. Whereas, among 6 bacterial strains isolated from dye-polluted effluents, 3 (AF1, CF3 and DF4) were strong biofilm formers.

### 3.2. Morphological and Biochemical Identification

The morphological characteristics of the bacterial strains are presented in Table S3. All the bacterial strains were Gram-positive. Morphologically, most of the bacterial colonies were circular, opaque, with an entire margin and smooth surface. The biochemical characterization of the bacterial strains is presented in Table S4. Biochemically, all strains (2A, 4A, 5A and 6B) were positive for the starch hydrolysis test. All were positive for urease, except for 5A. Two strains (4A and 5A) were positive for catalase, whereas 2A and 6B were catalase negative. Based on morphological and biochemical characterization, the isolates were identified up to the genus level. The findings indicated that most of the bacterial strains belonged to genus *Bacillus*, except for 4A, which belonged to the genus *Enterobacter* and AF1, which belonged to *Lysinibacillus.*

### 3.3. 16S rRNA Sequencing Study

**Figure 1:**
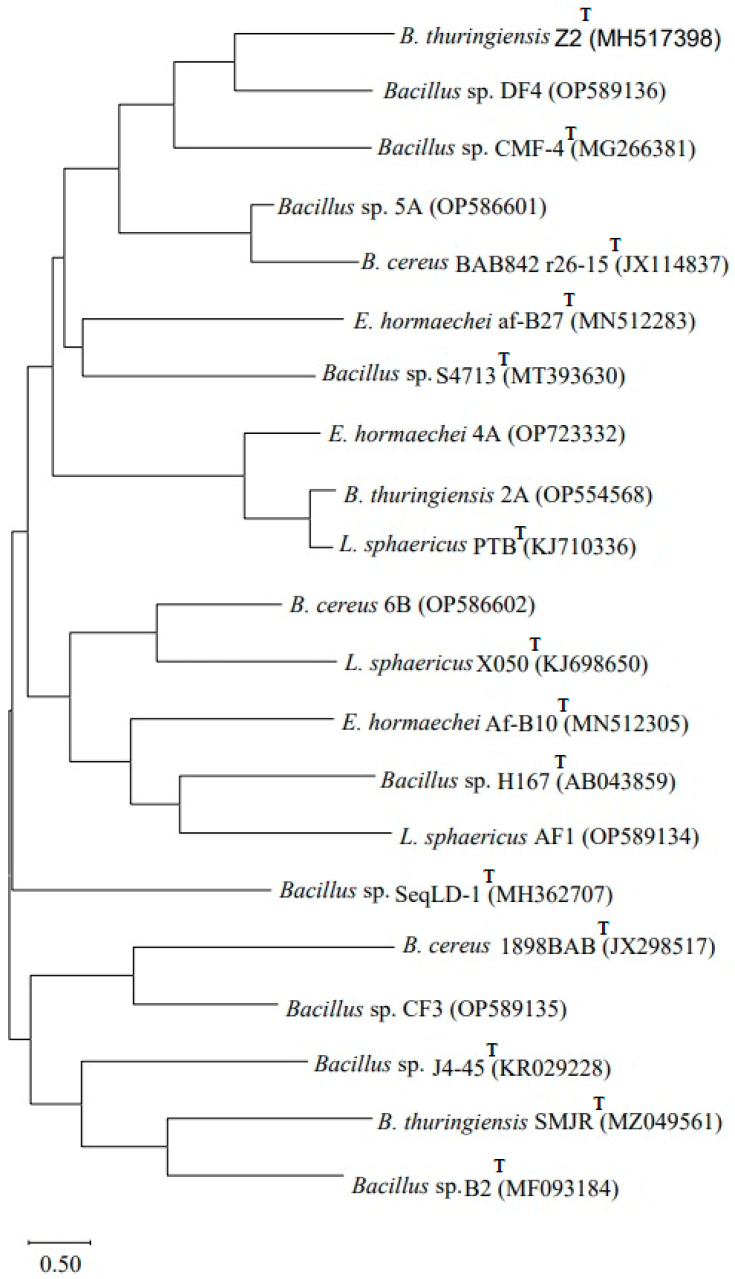
Phylogenetic tree based on 16S rRNA gene sequencing showing genetic variability among the seven biofilm-forming strains obtained from pesticides, dye-contaminated sites and type strains (T). The tree was constructed using MEGA X software by the neighbor-joining method.

The obtained sequences following 16S rRNA gene amplification were subjected to NCBI BLAST, which confirmed that *Bacillus* sp. (2A) showed 99% homology with *Bacillus thuringiensis* 2A (OP554568), *Enterobacter* sp. (4A) showed 100% homology with *Enterobacter hormaechei* 4A (OP723332), *Bacillus* sp. (5A) showed 100% homology with *Bacillus* sp. 5A (OP586601) and *Bacillus* sp. (6B) showed 99% homology with *B. cereus* 6B (OP586602). The strain *Lysinibacillus* (AF1) showed 99% homology with *L. sphaericus* AF1 (OP589134), *Bacillus* sp. strain (CF3) showed 99% homology with *Bacillus* sp. CF3 (OP589135) and *Bacillus* sp. (DF4) showed 100% homology with *Bacillus* sp. DF4 (OP589136). A phylogenetic tree (Figure 1) based on the 16S rRNA analysis was constructed using MEGA X software version 11 by the neighbor-joining tree method.

### 3.4. Time Kinetics of Biofilm Formation

**Figure 2:**
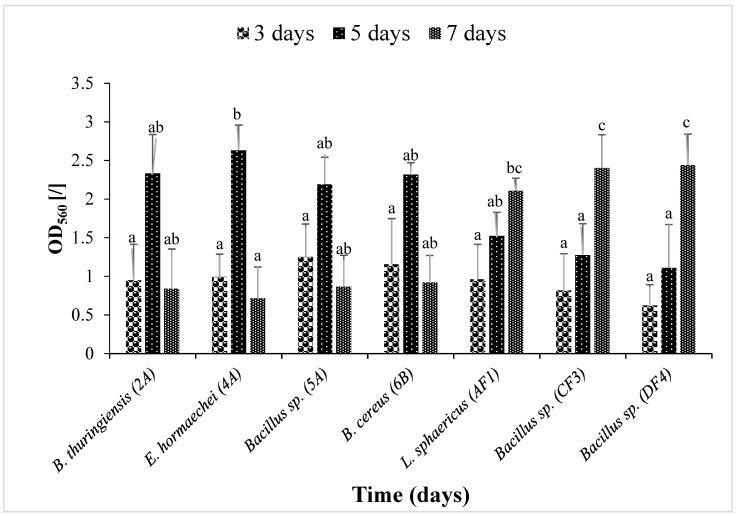
Biofilm time kinetics by seven isolated strains. Optical density (O.D_560_) was measured. Data are presented as mean ± SEM. Bars having no common superscript are significantly different at *p* < 0.05.

Figure 2 illustrates that the bacterial strains *B. thuringiensis* (2A), *E. hormaechei* (4A), *Bacillus* sp. (5A) and *B. cereus* (6B) showed significant (*p* ≤ 0.05) biofilm formation on the 5th day compared to *L. sphaericus* (AF1), *Bacillus* sp. (CF3) and *Bacillus* sp. (DF4), which were strong biofilm formers on the 7th day.

### 3.5. UV–VIS Spectroscopic Analysis of CYP and IMI before and after Biofilm Treatment

**Figure 3:**
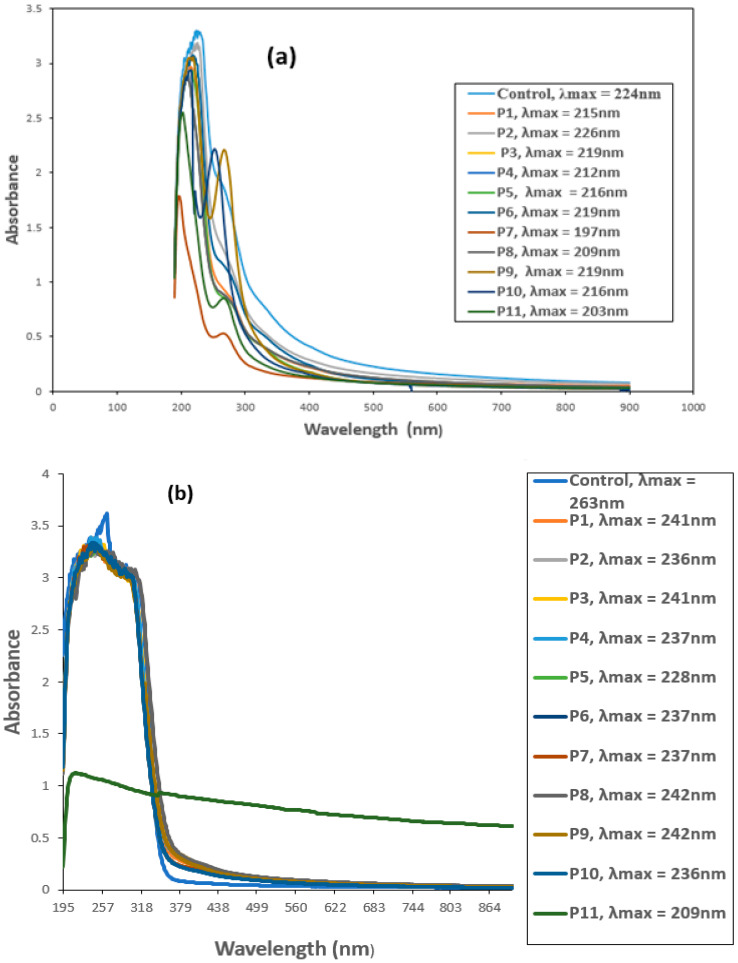
UV-VIS spectra of (**a**) Cypermethrin (CYP) and (**b**) Imidacloprid (IMI) before and after biofilm treatment. P1 = *B. thuringiensis*, P2 = *E. hormaechei*, P3 = *Bacillus* sp. (5A), P4 = *B. cereus*, P5 = *B. thuringiensis* + *E. hormaechei*, P6 = *B. thuringiensis* + *Bacillus* sp. (5A), P7 = *B. thuringiensis* + *B. cereus*, P8 = *E. hormaechei* + *Bacillus* sp. (5A), P9 = *E. hormaechei* + *B. cereus*, P10 = *Bacillus* sp. (5A) + *B. cereus*, P11 = *B. thuringiensis* + *E. hormaechei* + *Bacillus* sp. (5A) + *B. cereus*.

The biodegradation of CYP and IMI was monitored by observing changes in the UV–VIS absorption spectrum and comparing with the respective controls. Untreated CYP and IMI showed maximum absorption peaks at 224 and 263 nm, respectively, within the UV region (200–400 nm). This suggests that absorbance at 224 nm for CYP (Figure 3a) and 263 nm for IMI (Figure 3b) would effectively estimate IMI and CYP biodegradation.

**Figure 4:**
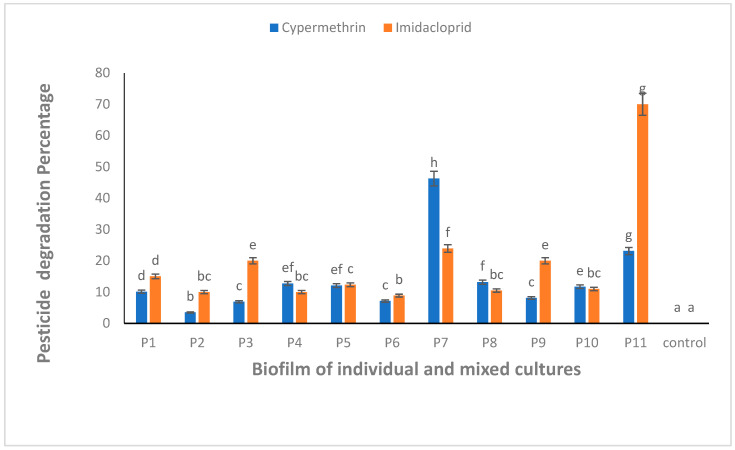
CYP and IMI degradation using biofilm of individual (P1, P2, P3 and P4) and mixed culture (P5, P6, P7, P8, P9, P10 and P11) isolates from pesticide-polluted soil. P1 = *B. thuringiensis*, P2 = *E. hormaechei*, P3 = *Bacillus* sp. (5A), P4 = *B. cereus*, P5 = *B. thuringiensis* + *E. hormaechei*, P6 = *B. thuringiensis* + *Bacillus* sp. (5A), P7 = *B. thuringiensis* + *B. cereus*, P8 = *E. hormaechei* + *Bacillus* sp. (5A), P9 = *E. hormaechei* + *B. cereus*, P10 = *Bacillus* sp. (5A) + *B. cereus*, P11 = *B. thuringiensis* + *E. hormaechei* + *Bacillus* sp. (5A) + *B. cereus*. Bars having no common superscript are significantly different at *p* < 0.05.

All the biofilm-forming bacteria obtained from the pesticide-polluted soil samples showed the potential to degrade CYP and IMI pesticides. The highest degradation (46.2%) against CYP was shown by a mixed culture of *B. thuringiensis + Bacillus* sp. (P7) (Figure 4). Similarly, IMI was highly degraded (70.0%) by mixed culture of all bacterial strains, *B. thuringiensis* + *E. hormaechei + Bacillus* sp. + *B. cereus* (P11) (Figure 4). One-way ANOVA followed by a post hoc Tukey test showed that the results of the biodegradation of CYP and IMI were statistically significant at *p* ≤ 0.05.

### 3.6. UV–VIS Spectroscopic Analysis of MG and CR before and after Biofilm Treatment

**Figure 5:**
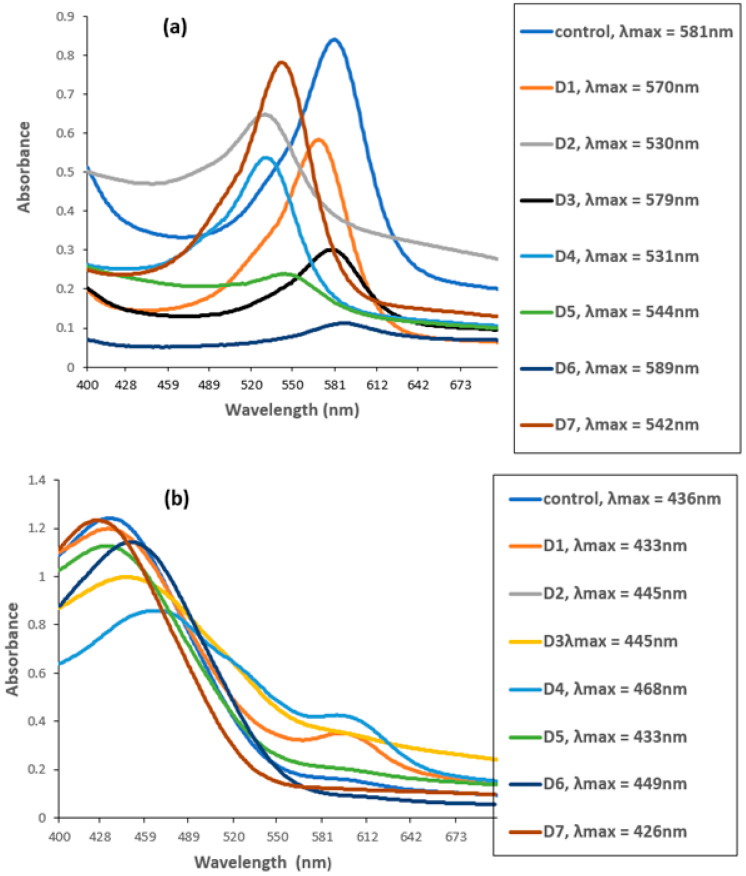
UV–VIS spectra of (**a**) Malachite green (MG) and (**b**) Congo red before and after biofilm treatment. D1 = *L. sphaericus*, D2 = *Bacillus* sp. (CF3), D3 = *Bacillus* sp. (DF4), D4 = *L. sphaericus* + *Bacillus* sp. (CF3), D5 = *L. sphaericus* +*Bacillus* sp. (DF4), D6 = *Bacillus* sp. (CF3) + *Bacillus* sp. (DF4), D7 = *L. sphaericus* + *Bacillus* sp. (CF3) + *Bacillus* sp. (DF4).

The UV–VIS spectrophotometer measured the degradation of CR and MG by biofilms of individual and mixed culture isolates. The untreated MG and CR showed major peaks at 581 nm (Figure 5a) and 436 nm (Figure 5b).

**Figure 6:**
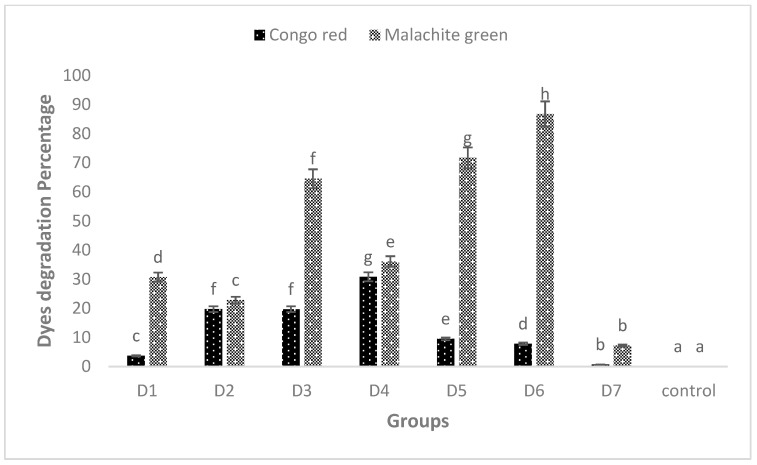
MG and CR degradation using biofilm of individual (D1, D2 and D3) and mixed culture (D4, D5, D6 and D7) biofilm. The data presented are mean ± standard errors of three independent experiments. D1 = *L. sphaericus*, D2 = *Bacillus* sp. (CF3), D3 = *Bacillus* sp. (DF4), D4 = *L. sphaericus* + *Bacillus* sp. (CF3), D5 = *L. sphaericus* + *Bacillus* sp. (DF4), D6 = *Bacillus* sp. (CF3) + *Bacillus* sp. (DF4), D7 = *L. sphaericus* + *Bacillus* sp. (CF3) + *Bacillus* sp. (DF4). Bars having no common superscript are significantly different at *p* < 0.05.

However, these peaks completely disappeared when the dyes were treated with biofilm of individual and mixed culture isolates. The decrease in absorbance indicated the successful degradation of MG and CR using the studied isolates. Among these, *Bacillus* sp. CF3 + *Bacillus* sp. DF4 (D6) showed the highest degradation (86.76%) against MG (Figure 6). Similarly, a mixed culture of *L. sphaericus + Bacillus* sp. (D4) showed only 30.78% degradation potential against CR dye (Figure 6). One-way ANOVA followed by a post hoc Tukey test showed that the results of the biodegradation of pesticides were statistically significant (*p* ≤ 0.05).

### 3.7. FTIR Analysis of CYP and IMI before and after Biodegradation

**Figure 7:**
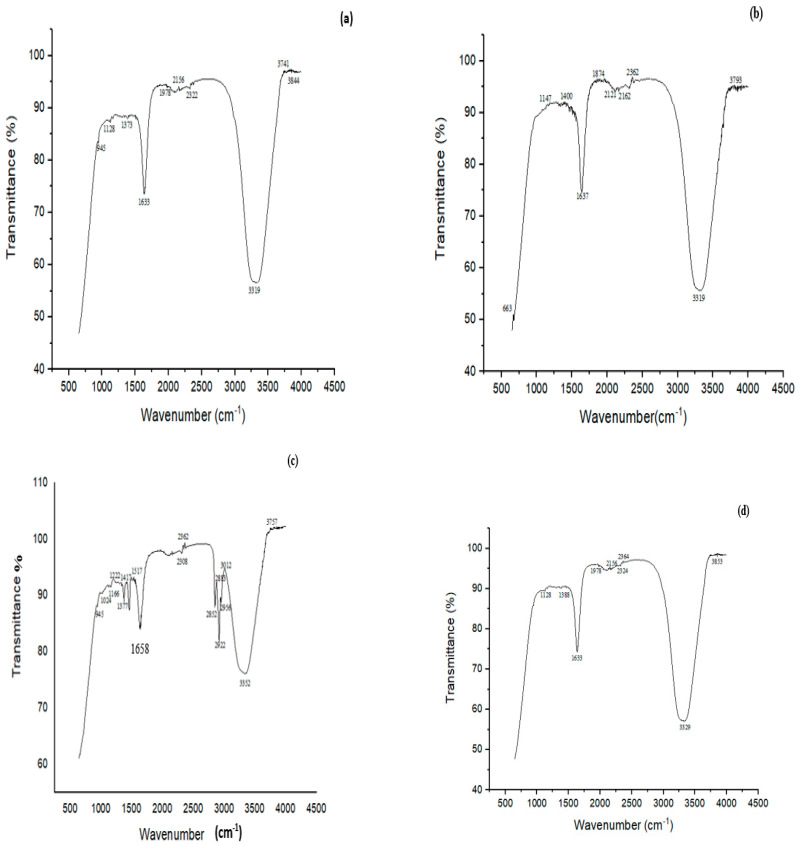
FTIR spectra of (**a**) CYP control, (**b**) CYP treated with a biofilm of mixed culture of *B. thuringiensis* + *Bacillus* sp. (5A) (P7), (**c**) Imidacloprid (IMI) control, (**d**) IMI treated with a biofilm of mixed culture of *B. thuringiensis* + *E. hormaechei + Bacillus* sp. (5A) + *B. cereus* (P11).

FTIR study showed many characteristic peaks at 945, 1128, 1373, 1633, 1978, 2156, 2322, 3319, 3741 and 3844 cm^−1^ in the controls (Figure 7a). These peaks signified various chemical bonds present in the structure of CYP. The band at 945 cm^−1^ was assigned to the asymmetric wagging vibrations of the terminal dihalovinyl group. The peak at 1373 cm^−1^ belonged to the N‒O symmetric stretch (nitro compound). The bands at 3319 and 1633 cm^−1^ corresponded to N-H and C=O amide groups, respectively. Treatment with a mixed culture of *B. thuringiensis* + *Bacillus* sp. (P7) resulted in the formation of five new peaks at 663, 1147, 1400, 1874, and 2121 cm^−1^, which belonged to the ‒CO group (C=O), ‒O‒ stretching, deformation of CH_2_ in the R‒CH_2_‒CN structure, asymmetric carbonyl stretching and the aryl‒O of diphenyl ether, which confirmed the successful degradation of CYP (Figure 7b).

The FTIR spectrum of control (IMI) displayed peaks at 945, 1024, 1166, 1222, 1377, 1417, 1517, 1658, 2308, 2362, 2852, 2956, 3012, 3352 and 3757 cm^−1^ (Figure 7c). A characteristic peak at 1166 cm^−1^ corresponded to N-O for banding of the azoxy compound, 1222 cm^−1^ for the vibration band of C=N in the pyridine ring and 1377 cm^−1^ for NO_2_ stretching of the compound in IMI, 1417 for ‒CH= aromatic rings, and 1517 cm^−1^ for the vibration band of N=N in the imidazolidine. The peak at 3352 cm^−1^ depicted the O‒H stretching vibration. The FTIR spectrum of biodegraded IMI with *B. thuringiensis* + *E. hormaechei + Bacillus* sp. + *B. cereus* (P11) showed an alteration in the FTIR spectrum compared to the control. Many peaks (i.e., 1024, 1417 and 1517 cm^−1^) present in the control completely disappeared after degradation by the P11 biofilm (*B. thuringiensis* + *E. hormaechei* + *Bacillus* sp. (5A) + *B. cereus)*. The disappearance of the peak at 1517 cm^−1^ in the spectrum by the all-strains mixed culture (P11) associated with the vibration band of N=N in the imidazolidine showed the degradation of IMI (Figure 7d).

### 3.8. FTIR Analysis of MG and CR before and after Biodegradation

**Figure 8:**
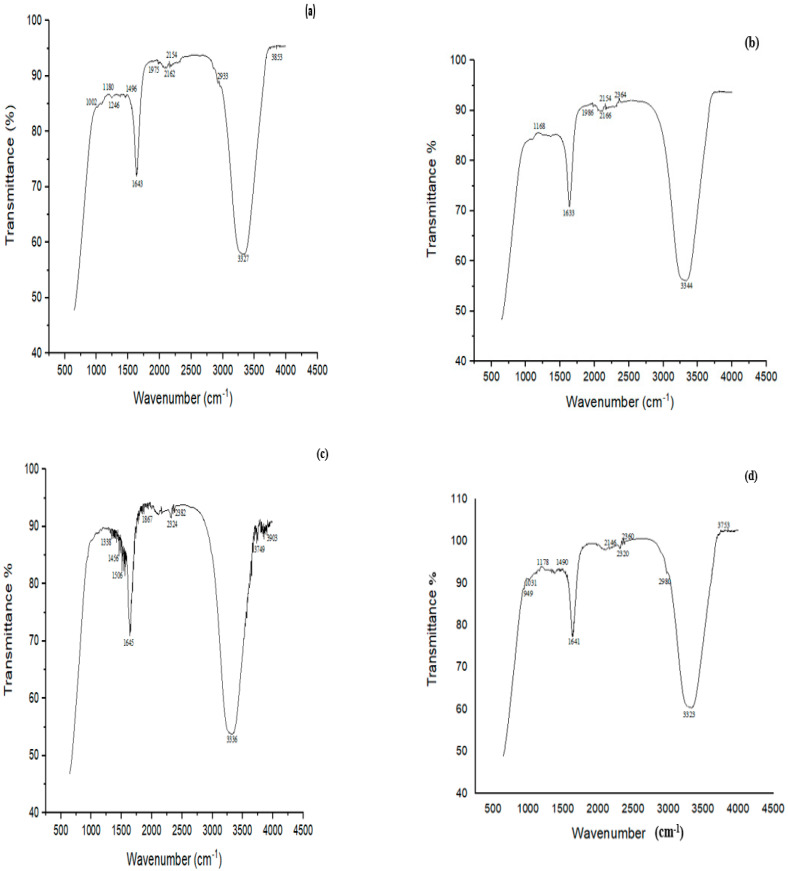
FTIR spectra of (**a**) MG control, (**b**) MG treated with biofilm of *Bacillus* sp. (CF3) + *Bacillus* sp. (DF4) (D6) (**c**) CR control, (**d**) CR treated with a biofilm of mixed culture of *L. sphaericus + Bacillus* sp. (CF3) (D4).

FTIR analysis of MG before treatment showed characteristic peaks at 3800–3100 cm^−1^ indicating NH stretching vibrations (Figure 8a). The peak at 3327 cm^−1^ was related to H-bonded NH symmetric stretching. The peaks at 1246 cm^−1^ and 1496 cm^−1^ were ascribed to in-phase and out-of-phase deformation vibrations of the CH_3_ group, respectively. The peak at 1180 cm^−1^ was attributed to C‒N stretching vibrations, and that at 2933 cm^−1^ showed C‒H asymmetric stretching. When MG was treated with biofilm of *Bacillus* sp. (CF3) + *Bacillus* sp. (DF4) (D6), many peaks disappeared, such as a peak at 1002 cm^−1^ associated with C-N stretching vibrations, suggesting MG degradation (Figure 8b).

FTIR analysis of CR dye revealed bands at 1338, 1456, 1506, 1645, 1867, 2324, 2382, 3336, 3749 and 3903 cm^−1^ (Figure 8c). The peak at 3336 cm^−1^ was attributed to the presence of N-H, 1456 cm^−1^ to C‒H, 1338 cm^−1^ to C‒N bending vibrations, and 1506 cm^−1^ to N = N stretching vibrations. The study’s principal characteristic peaks were deformed, or partly or completely disappeared following the biodegradation experiment. The cleavage of the azo bond was confirmed by the absence of N = N stretching vibrations at 1506 cm^−1^ of the biodegraded CR following treatment with *L. sphaericus + Bacillus* sp. (D4) (Figure 8d)**.**

### 3.9. TLC Analysis of CYP, IMI, MG and CR before and after Biodegradation

The comparison of TLC chromatograms before and after degradation showed the appearance of additional bands of different retention factor (*R_f_*) values compared to the control *R_f_* value. This clearly indicated the degradation of pesticides and dyes into intermediate products. The *R_f_* value of CYP after degradation with the biofilm of a mixed culture of *B. thuringiensis* + *Bacillus* sp. (P7) demonstrated the formation of two metabolites with distinct *R_f_* values, e.g., 0.47 and 0.71, compared to the control. The *R_f_* values of these two metabolites matched with 3-phenoxybenzaldehyde (3-PBA) and phenol as standard. An *R_f_* value 0.33 of IMI (control) was observed. The biodegraded metabolites of IMI after treatment with a biofilm of a mixed culture of *B. thuringiensis* + *E. hormaechei + Bacillus* sp. + *B. cereus* (P11) showed the appearance of three spots with *R_f_* values 0.21, 0.34 and 0.76. The spots at 0.21, 0.34 and 0.76 showed the presence of IMI, 6-chloronicotinic acid and other intermediate metabolites in the degraded sample. TLC analysis of MG treated with a biofilm of a mixed culture of *Bacillus* sp. (CF3) + *Bacillus* sp. (DF4) (D6) showed that the sample had two more spots at *R_f_* values 0.65 and 0.72 compared to the control (0.55). The *R_f_* value of CR (control) was 0.22. The metabolites formed after treatment with a biofilm of a mixed culture of *L. sphaericus + Bacillus* sp. (CF3) (D4) showed two additional spots with *R_f_* values of 0.35 and 0.48 (Figure S2).

## 4. Discussion

The biofilm-mediated biodegradation of pollutants is a novel and attractive approach to protect our environment from pesticide and dye pollution. Microbes hide and protect themselves under a matrix of biofilm and produce various enzymes to degrade pollutants [23,44]. A variety of pollutants, including hormones, phenolics, pharmaceuticals, and toxic compounds, even in low concentrations, can be bound to binding sites and consumed by cells in the biofilm [45]. In the current study, indigenous biofilm-forming bacteria were isolated to assess their ability to degrade pesticides and dyes. The best results for the isolation of pesticides and dye-degrading bacteria are achieved from samples collected from contaminated fields with insecticides, dyes and other chemicals. The ability to produce biofilm was assessed on CR agar. Application of CR agar is a widely used method to check qualitatively the biofilm-forming potential of bacterial strains [46].

The crystal violet staining method revealed that, among all the strains, four strains from pesticide-contaminated sites showed mature biofilm formation on the 5th day compared to strains obtained from dye effluents, which produced maximum biofilm on the 7th day of incubation. These results are supported by Shukla and Rao, who isolated bacterial strains and used the crystal violet method in a biofilm time kinetics study [47]. Based on morphological, biochemical and genetic characterization, the strains found belonged to the species *Bacillus*, *Lysinibacillus* and *Enterobacter*. *Bacillus* strains were reported to possess biodegradation potential of soil pollutants [48]. *B. cereus* was found to be the most promising strain in the current study. Previously, it was observed to destroy the reactive textile dye Novacron Super Black G [49].

*B. thuringiensis* is known for its wide use in biological control [50]. Many researchers have reported many genera of *Bacillus* that show potential for degrading various pesticides and other xenobiotic compounds [51]. In recent years, microbial biodegradation has become more popular due to the fact that pesticides are mostly microbial nutrients that breakdown into tiny molecules such as CO_2_ and H_2_O. The process has been understood to be an enzymatic reaction, involving the compound entering in a specific way inside microbes and then going through a series of physiological and biochemical reactions under the action of various enzymes until the pesticide is completely degraded or broken down into smaller molecular compounds with non-toxicity or low toxicity [52]. Pesticide-stress-sensitive enzymes, including esterase aldehyde dehydrogenase and laccase, have previously been discovered in the genus *Bacillus*, and can breakdown organophosphate, pyrethroid, carbamate pesticides and organochlorine pesticides. The aldehyde dehydrogenase oxidize aldehyde group plays a critical role in the degradation of cypermethrin [53]. The *B. cereus* genome encodes a laccase gene which may be activated under stress conditions. This regulatory gene is known for the biodegradation of both CYP and IMI [54] and might be responsible for the efficient biodegradation ability shown by the *Bacillus* strains in the current study.

Similarly, in CYP-resistant bacteria, enzymes such as malate dehydrogenase, α-ketoglutarate semialdehyde dehydrogenase, glycerol-3-phosphate dehydrogenase, and formate dehydrogenase were downregulated. All the enzymes stated above play a significant role in the tri-carboxylic acid cycle, implying that bacteria may have taken up CYP via new metabolites for growth. The authors have already reported that, under stress conditions, the percentage of stress-sensitive proteins and pesticide-degrading proteins was high, making the organism more comfortable [41]. Similarly, in another study, genomic, transcriptome, and proteomic studies of *B. cereus* GW-01 during β-cypermethrin and its metabolite degradation revealed that α/β- hydrolase and a cytochrome aa3 quinol oxidase were responsible for β-cypermethrin and 3-phenoxy benzoic acid (3-PBA) degradation, respectively [42].

Following Haque et al. [43], the biodegradation potential of pesticides by bacterial biofilms was analyzed both individually and in combination. Analytical techniques, i.e., UV–VIS, FTIR and TLC, were used for confirmation of the biodegradation of CYP, IMI, MG and CR. Similar to this study, Kalyani et al. [43] used the same analytical techniques to confirm the biodegradation of reactive textile dye red BL1 by an isolated bacterium *Pseudomonas* sp. SUK1. The optical density was observed at 224 nm for CYP using a UV–VIS spectrophotometer. Similarly, Kaur et al. [55] reported that UV–VIS spectrophotometry showed absorbance at 220 nm for CYP. The optical density for IMI was recorded at 263 nm. Similar results were reported by Kitsiou et al. [45] on the characteristic absorbance of IMI at 270 nm within the UV region [56]. CYP was degraded up to 46.2% following treatment with a mixed culture of *B. thuringiensis* + *Bacillus* sp. (P7), while IMI was significantly degraded (70%) by mixed bacterial biofilms, *B. thuringiensis* + *E. hormaechei* + *Bacillus* sp. + *B. cereus* (P11). This is comparable to findings by Anhalt et al. [57] who reported strain PC-21 with 37 to 58% degradation ability of 25 mgL^−1^ IMI over a period of three weeks. Similarly, Akoijam isolated 50 soil bacteria, among these *Bacillus* sp. was able to degrade 45.48% of the initial amount of IMI at a concentration of 25 mgL^−1^ [58]. Gupta et al. [28] reported *Pseudomonas* sp. RPT 52, isolated from an agricultural field with 46.5% degradation ability of IMI over a period of 40 h. *Leifsonia* sp. PC-21 isolated from soil was reported to degrade 37–58% of IMI in full-strength tryptic soy broth [59]. Tiwari et al. [60] reported use of the *Tepidibacillus decaturensis* strain ST1 resulted in the degradation of around 77.5% and 85% of IMI in sterile and unsterile soils, respectively, within 45 days. Madhuban et al. [61] investigated the biodegradation of IMI by an aerobic bacterium capable of digesting IMI isolated from an agricultural field soil using enrichment culture. The authors reported the strain as *Burkholderia cepacia* (strain CH9) on a mineral-salt medium supplied with 50 g mL^−1^ of IMI having 69% IMI degradation ability within 20 days.

The current study findings of IMI degradation are much better than those reported by other researchers, especially with respect to the time period required to achieve maximum degradation. To the best of our knowledge, this is the first report that demonstrates the biodegradation potential of *E. hormaechei* against IMI. The degradation ability of P7 and P11 biofilm for CYP and IMI, respectively, shows that these bacterial biofilms could be useful in the effective management of pesticide-polluted soils.

UV–VIS analysis of dyes revealed that MG showed a major peak at 581 nm and CR at 436 nm. A mixed culture of *Bacillus* sp. + *Bacillus* sp. (D6) showed significantly highest degradation (86.76%) potential of MG compared to *L. sphaericus* + *Bacillus* sp. (D4), which degraded CR by 30.78% only. Maximum MG dye degradation by *Enterobacter* sp. at pH 7 and temperature 37 °C was reported, which was similar to the pH and temperature of this study [62]. This indicated the relatively better potential of D6 for MG degradation. MG is hard to remove from aqueous solutions and is poisonous to most major bacteria due to its characteristics. Adsorption is the main topic of study in bioremediation. There has been a brief investigation into the biodegradation of MG due to MG’s toxicity to microorganisms. Previously, the authors utilized crude enzymes or crude extracts for MG degradation [63]. The current study confirmed a biofilm of mixed culture (D6) has strong potential for MG degradation.

However, information regarding the uses of a mixed culture biofilm to degrade and detoxify the CR is very limited in the literature [42,64,65]. To the best of our knowledge, biofilms of an isolate *L. sphaericus* capable of degrading CR are little documented in the literature. Our report showed that a biofilm of *L. sphaericus* can degrade CR. In other studies, *L. sphaericus* was used to biodegrade petroleum hydrocarbon and biosurfactants [66]. Earlier, the decolorization efficiency of Reactive Yellow F3R and Joyfix Red RB dyes by textile effluent non-adapted *L. sphaericus* MTCC 9523 in optimized operational conditions was investigated [67]. This biodegradation of dyes may be due to the presence of several dye-degrading enzymes, such as azo reductases, laccases, veratryl alcohol oxidase, tyrosinases and peroxidases naturally present in *Bacillus* sp., aiding the biodegradation process under standard conditions [67]. Bacteria can generate peroxidase, which can destroy azo dyes [43]. The decolorization of azo dyes by biofilm-producing *Bacillus* sp. was already reported [68]. UV–VIS analysis as a nondestructive method enables real-time measurement [69].

Sinha et al. [51] reported that various endogenous bacteria adapt themselves to dye-contaminated wastewater and degrade azo dyes. A horizontally transportable microbial genetic pool has been described to play a substantial part in stress adaptation. The horizontal transfer of genes helps in the fast microbial degradation of xenobiotic substances, and the genes of degradative enzymes are transported by mobile genetic elements, like transposons and plasmids, or they may be contained within the chromosome itself [70].

FTIR confirmed the degradation of CYP, IMI, MG and CR by bacterial biofilms. The FTIR spectrum of CYP (control) showed a peak at 1373 cm^−1^ linked with ether-cyanate that was not detected after degradation with P7. A slight increase in 1637 cm^−1^ associated with carbonyl signals was noted, suggesting CYP biodegradation. The results of this study are very comparable with those demonstrated by Sharma et al. [32] with similar peaks for CYP biodegradation by *Bacillus* sp. (SG2) isolated from CYP-polluted sites. Similar to this study, asymmetric carbonyl stretching, C=C stretching of the aromatic rings, CH_2_ deformation in the R‒CH_2_‒CN structure, and (C=O)‒O‒ stretching were reported by Armenta et al. and Kaur et al. using *Fusarium* sp. [55,71]. CYP was possibly degraded through cleavage at the ester bond, releasing many intermediate compounds [72,73].

In the FTIR spectra of IMI (control), peaks at 1517, 1430 and 1377 cm^−1^ associated with the vibration band of N=N in the imidazolidine, ‒CH=aromatic rings and NO_2_ stretching of the compound, respectively, were observed. Likewise, Negi et al. [74] reported peaks at 1570, 1450 and 1430 cm^−1^ in the control (IMI), which corresponded to N=N in the imidazolidine, ‒CH= aromatic rings and NO_2_ stretching, respectively. The peaks at 1166, 1222 and 3352 cm^−1^ corresponded to N‒O for banding of the azoxy compound, C=N of the pyridine ring, and O-H stretching, respectively. Phugare et al. [11] made similar observations when determining the changes in the biodegradable sample compared to the control. FTIR analysis suggested the MG peak at 1180 cm^−1^ could be ascribed to C‒N stretching vibrations; 2933 cm^−1^ showed C-H asymmetric stretching. Similar to this study, C-N stretching at 1170 cm^−1^ and C-H stretching of asymmetric ‒CH_3_ at 2923 cm^−1^ corresponded to MG [17]. FTIR analysis of CR revealed C-N bending vibrations at 1338 cm^−1^ and N=N stretching vibrations at 1506 cm^−1^. Similarly, the disappearance of the peak at 1578 cm^−1^ represented N=N stretching vibrations for biodegraded CR [75]. The disappearance of N=N indicated the azo bond’s cleavage by the mixed culture biofilm [76].

The degradation of CYP, IMI, MG and CR was further supported by TLC. The spots noticed prior to degradation differed significantly from those observed with the supernatant acquired after degradation. The pesticides and dyes before degradation were very different from the supernatant obtained following degradation. Different *R_f_* values obtained in the TLC experiment confirmed the degradation of pesticides and dyes into intermediate products. CYP after degradation showed two additional spots at *R_f_*: 0.47 and *Rf*: 0.71. Relatively similar band patterns of 3PBA and phenol on TLC were observed in the CYP degradation by *B. cereus* [77]. Three spots of distinct *R_f_* values 0.21, 0.34 and 0.76 appeared after IMI degradation. In a previous study, similar bands of IMI and 6-chloronicotinic acid on TLC were observed in the microbial degradation of IMI in the silkworm [11]. The TLC results of degraded MG and CR showed two additional spots with different *R_f_* values compared to the control. The crucial phase in bacterial dye degradation is the reductive cleavage of the N=N (azo) bond, which results in the generation of colorless aromatic amines. These amines undergo reduction to simpler forms, which are then oxidized [78].

In our study, the biofilms of mixed culture appeared more competent than biofilms of individual cultures in selected pesticide and dye degradation. Furthermore, it was confirmed that single strain biofilms could also degrade, but the degradation efficiency was not as marked as for biofilms formed by mixed bacterial strains. Each bacterial strain in a bacterial consortium was demonstrated to degrade the dye molecule differently or to use the metabolites produced by the co-existing strain for additional breakdown [79]. Thus, the degradation rate of a bacterial consortium is usually higher than that of a single bacterial strain. More studies are required to identify biofilms of bacterial consortia capable of degrading other pesticides and/dyes into less toxic metabolites, followed by identification of the responsible genes and the in-depth mechanisms.

## 5. Conclusions

The current study investigated the biodegradation of CYP, IMI, MG, and CR using indigenous bacterial biofilms individually and in mixed consortia. Indigenous microbes under stress conditions can change their genetic profile and induce mutations, which make them adapted to different environmental conditions reflecting their biochemical metabolism diversity. In our investigation, mixed culture biofilms degraded pesticides and dyes more effectively than individual isolates. Briefly, biofilms of mixed culture bacteria can be applied in the biodegradation of pesticides and dye-polluted soil and effluents. It is recommended that biofilm-forming strains could be further investigated for the extraction of bioactive components and genes/enzymes for their ability to degrade other hazardous pollutants to obtain a broad-spectrum degradation approach.

## Data Availability

All the data is available in the manuscript.

## Supplementary Materials

The following supporting information can be downloaded at: https://www.mdpi.com/article/10.3390/microorganisms11092163/s1, Figure S1: FTIR spectra of biodegraded CYP, IMI, MG and CR. (a) CYP after treatment with biofilm of *B. thuringiensis* (g1), (b) CYP after treatment with biofilm of *E. hormaechei* (g2), (c) CYP after treatment with biofilm of *Bacillus* sp. (5A) (g5), (d) CYP after treatment with biofilm of *B. cereus* (g4), (e) CYP after treatment with mixed culture of *B. thuringiensis* + *E. hormaechei* + *Bacillus* sp. (5A) + *B. cereus* (g11), (f) IMI after treatment with biofilm of *B. thuringiensis* (g1), (g) IMI after treatment with biofilm of *E. hormaechei* (g2), (h) IMI after treatment with biofilm of *Bacillus* sp. (5A) (g3), (i) IMI after treatment with biofilm of *B. cereus* (g4), (j) IMI after treatment with biofilm of mixed culture of *B. thuringiensis* + *Bacillus* sp. (g7), (k) MG treated with biofilm of *L. sphaericus* (g1), (l) MG treated with biofilm of *Bacillus* sp. (CF3) (g2), (m) MG treated with biofilm of *Bacillus* sp. (DF4) (g3), (n) CR treated with biofilm of *L. sphaericus*, (g1), (o) CR treated with biofilm of *Bacillus* sp. (CF3) (g2), (p) CR treated with biofilm of *Bacillus* sp. (DF4) (g3).; Figure S2: Thin layer chromatography analysis of (a) CYP before and after biodegradation with P7, (b) IMI before and after degradation with P11, (c) MG before and after degradation with D6, (d) CR before and after degradation with D4. d- CYP, d-IMI, d-MG, d-CR = degraded CYP, degraded IMI, degraded MG and degraded CR, respectively. P7 = *B. thuringiensis* + *B. cereus*, P11 = *B. thuringiensis* + *E. hormaechei* + *Bacillus* sp. (5A) + *B. cereus*, D6 = *Bacillus* sp. (CF3) + *Bacillus* sp. (DF4) and D4 = *L. sphaericus* + *Bacillus* sp. (CF3); Table S1: Biofilm-forming bacterial isolates alone and in combination for pesticide and dye biodegradation. Table S2: Screening of biofilm producers from isolates obtained from pesticides and dye-contaminated effluents; Table S3: Morphological characteristics of bacterial isolates; Table S4: Biochemical characterization of bacterial isolates.
