# Supplementary material for: In Vitro Biofilm-Mediated Biodegradation of Pesticides and Dye-Contaminated Effluents Using Bacterial Biofilms
Source: Microorganisms. 2023 Aug 26;11(9):2163. doi: 10.3390/microorganisms11092163 (PMC10535849; doi:10.3390/microorganisms11092163)
Supplement: Supplementary file 1 [file microorganisms-11-02163-s001.zip › microorganisms-2524356-supplementary.pdf]

Table S1. Biofilm forming bacterial isolates alone and in combination for pesticides and dyes biodegradation

Groups	Biofilm forming bacterial isolates alone and in combination with each other	Test Compounds
P1	*B. thuringiensis* 2A	
P2	*E. hormaechei* 4A	
P3	*Bacillus* sp. 5A	**Pesticides**
P4	*B. cereus* 6B	(Cypermethrin
P5	*B. thuringiensis* 2A + *E. hormaechei* 4A	and
P6	*B. thuringiensis* 2A + *Bacillus* sp. 5A	Imidacloprid)
P7	*B. thuringiensis* 2A + *B. cereus* 6B	
P8	*E. hormaechei* 4A + *Bacillus* sp. 5A	
P9	*E. hormaechei* 4A + *B. cereus* 6B	
P10	*B. cereus* 6B + *Bacillus* sp. 5A	
P11	*B. thuringiensis* 2A + *E. hormaechei* 4A + *Bacillus* sp. 5A + *B. cereus* 6B	
D1	*L. sphaericus* AF1	
D2	*Bacillus* sp. CF3	
D3	*Bacillus* sp. DF4	**Dyes**
D4	*L. sphaericus* AF1 + *Bacillus* sp. CF3	(Malachite Green
D5	*L. sphaericus* AF1 + *Bacillus* sp. DF4	and Congo Red)
D6	*Bacillus* sp. CF3 + *Bacillus* sp. DF4	
D7	*L. sphaericus* AF1 + *Bacillus* sp. CF3 + *Bacillus* sp. DF4	

Table S2. Screening of biofilm producers from isolates obtained from pesticides and dyes contaminated effluents

Bacteria strains	Biofilm producer	Bacterial strains	Biofilm producers
2A	+	6A	_
2B	_	6B	+
3A	_	AF1	+
3B	_	BF2	_
4A	+	CF3	+
4B	_	DF4	+
5A	+	EF5	_
5B	_	FF6	_

+ = Positive (biofilm producer, showed black colour); − = negative (non- biofilm producer, red in colour)

Table S3. Morphological characteristics of bacterial isolates

Characteristics	2A	4A	5A	6B	AF1	CF3	DF4
Gram Staining	Positive	Positive	Positive	Positive	Positive	Positive	Positive
Shape	Rod	Circular	Circular	Rod	Circular	Circular	Rod
Color	Dark brown	Red	Cream	Dark brown	White	Yellowish	Off-white
Margin	Entire	Entire	Entire	Entire	Entire	Entire	Entire
Elevation	Raised	Raised	Umbonate	Complex	Flat	Raised	Flat
Opacity	Opaque	Opaque	Opaque	Opaque	Opaque	Opaque	Opaque
Surface	Smooth	Smooth	Smooth	Smooth	Smooth	Smooth	Smooth

Table S4. Biochemical characterization of bacterial isolates

Tests	2A	4A	5A	6B	AF1	CF3	DF4
Starch	+	+	+	+	-	-	-
Urease	+	+	-	+	-	-	-
Catalase	-	+	+	-	+	+	+
Glucose	+	+	+	+	+	-	-
Sucrose	+	+	+	+	-	-	-
Lactose	+	+	+	+	-	+	+
Methyl-red	+	+	+	+	-	+	-
Voges-Proskauer	+	+	+	+	-	+	-

+ = Positive; − = negative for each test

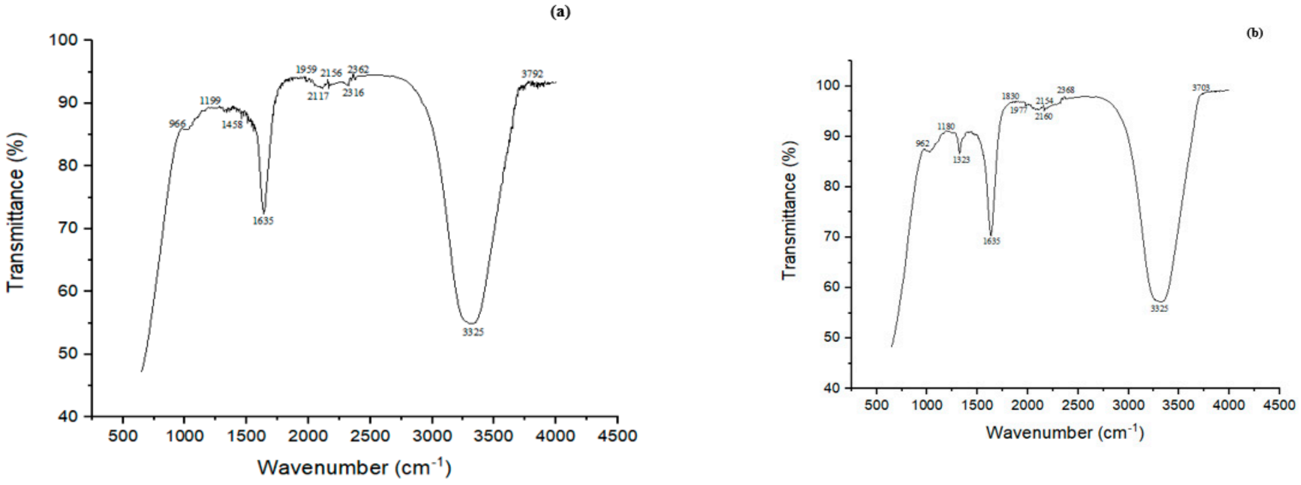

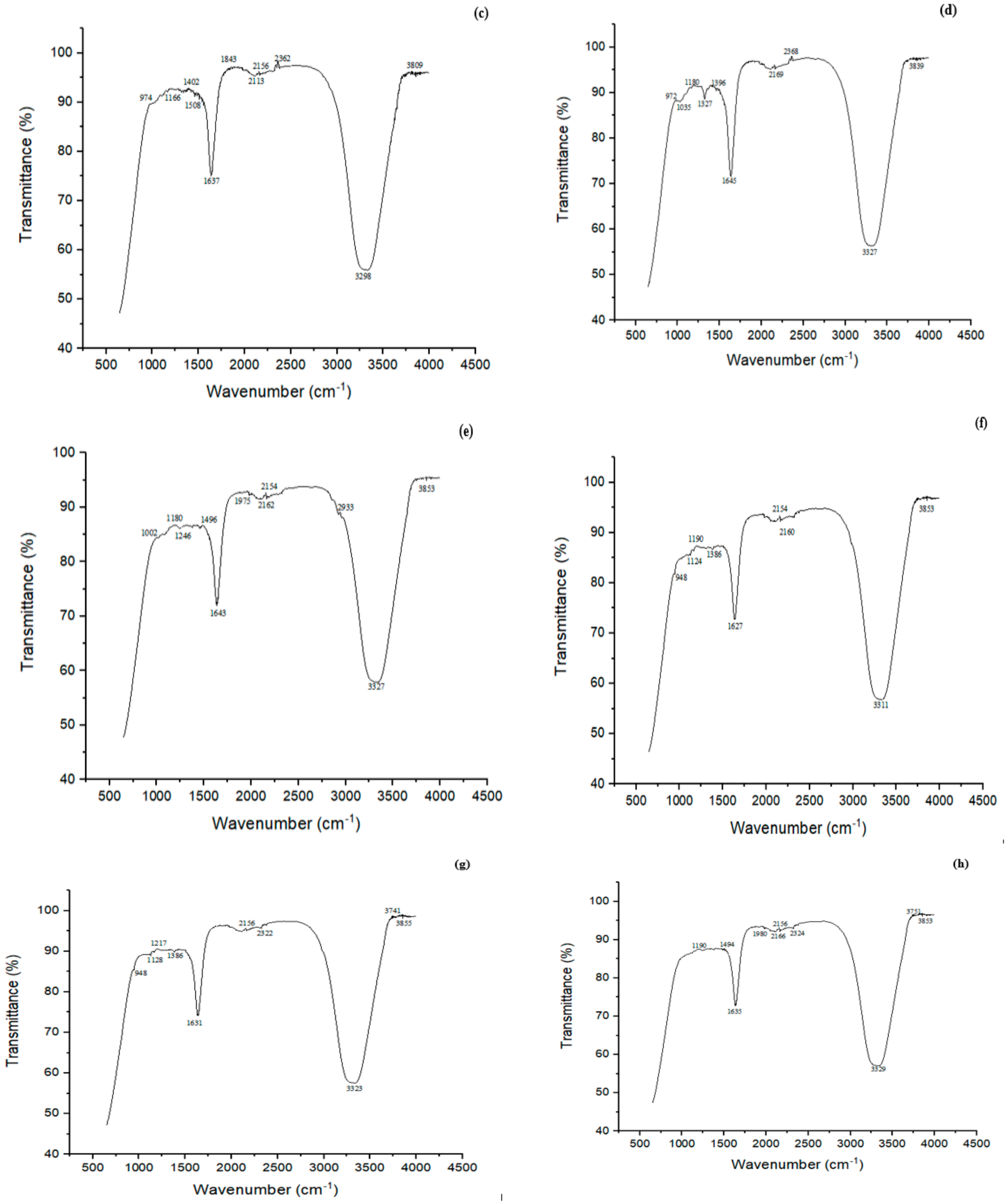
(c)
Transmittance (%)
Wavenumber (cm-1)
974
1166
1402
1508
1637
1843
2156
2113
2362
3298
3809
(d)
972
1035
1180
1327
1396
1645
2169
2368
3327
3839
(e)
1002
1180
1246
1496
1643
1975
2154
2162
2933
3327
3853
(f)
948
1190
1124
1386
1627
2154
2160
3311
3853
(g)
948
1217
1128
1386
1631
2156
2322
3323
3741
3855
(h)
1190
1494
1635
1980
2156
2166
2324
3329
3751
3853
100
90
80
70
60
50
40
500
1000
1500
2000
2500
3000
3500
4000
4500

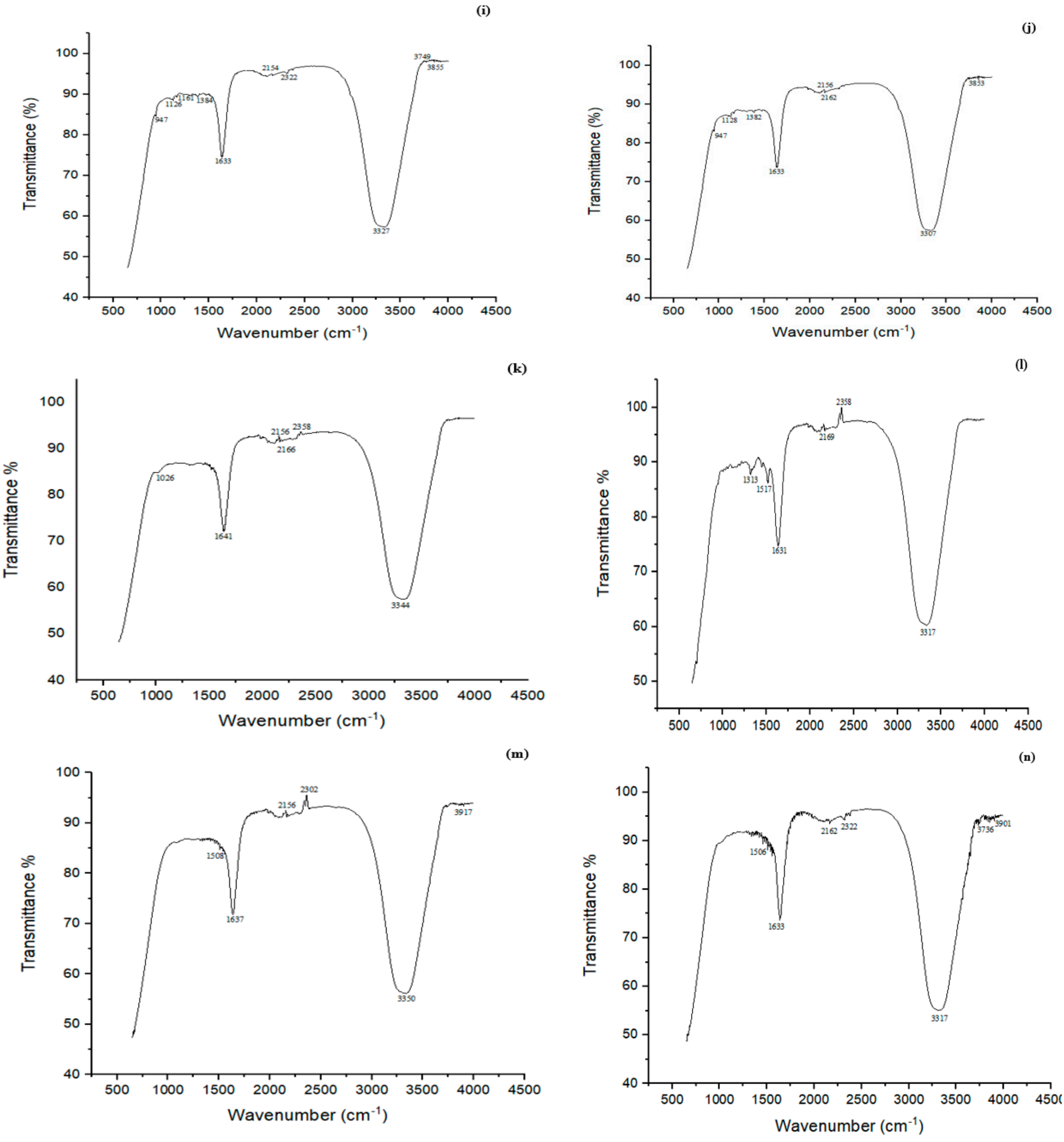
(i)
Transmittance (%)
Wavenumber (cm-1)
947
1126
1161
1384
1633
2154
2322
3327
3749
3855
(j)
Transmittance (%)
Wavenumber (cm-1)
947
1128
1382
1633
2156
2162
3307
3853
(k)
Transmittance %
Wavenumber (cm-1)
1026
1641
2156
2358
2166
3344
(l)
Transmittance %
1313
1517
1631
2169
2358
3317
(m)
Transmittance %
Wavenumber (cm-1)
1508
1637
2156
2302
3350
3917
(n)
Transmittance %
Wavenumber (cm-1)
1506
1633
2162
2322
3317
3736
3901

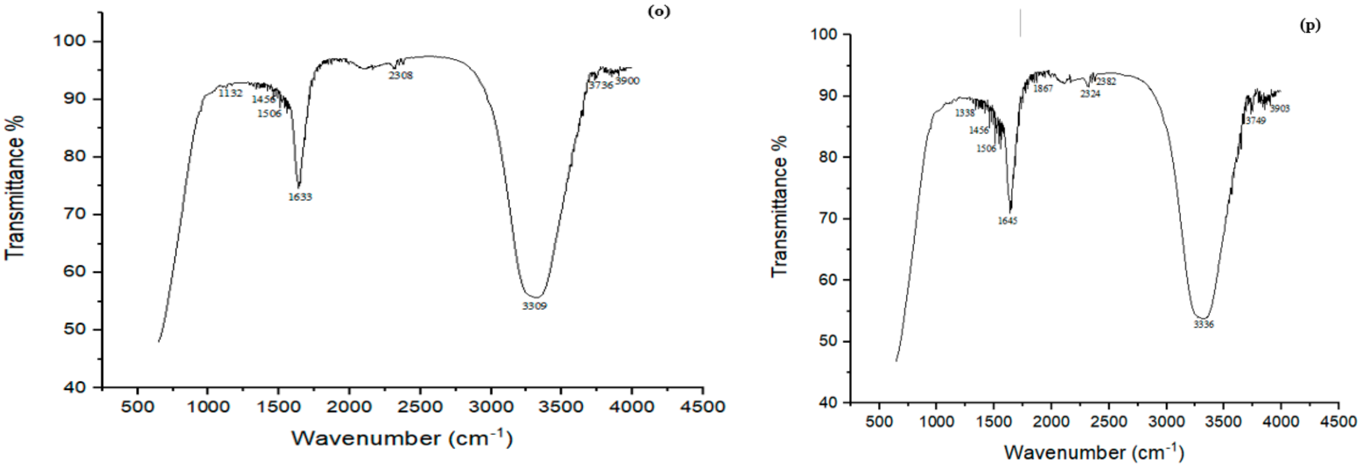

Figure S1. FTIR spectra of biodegraded CYP, IMI, MG and CR. (a) CYP after treatment with biofilm of *B. thuringiensis* (g1) (b). CYP after treatment with biofilm of *E. hormaechei* (g2), (c) CYP after treatment with biofilm of *Bacillus* sp. (5A) (g5), (d) CYP after treatment with biofilm of *B. cereus* (g4) (e). CYP after treatment with mixed culture of *B. thuringiensis*. + *E. hormaechei* + *Bacillus* sp. (5A) + *B. cereus* (g11). (f). IMI after treatment with biofilm of *B. thuringiensis* (g1) (g). IMI after treatment with biofilm of *E. hormaechei* (g2) (h). IMI after treatment with biofilm of *Bacillus* sp. (5A) (g3) (i). IMI after treatment with biofilm of *B. cereus* (g4) (j). IMI after treatment with biofilm of mixed culture of *B. thuringiensis* + *Bacillus* sp. (g7) (k). MG treated with biofilm of *L. sphaericus* (g1), (l). MG treated with biofilm of *Bacillus* sp. (CF3) (g2), (m). MG treated with biofilm of *Bacillus* sp. (DF4) (g3), (n). CR treated with biofilm of *L. sphaericus*, (g1), (o). CR treated with biofilm of *Bacillus* sp. (CF3) (g2), (p). CR treated with biofilm of *Bacillus* sp. (DF4) (g3).

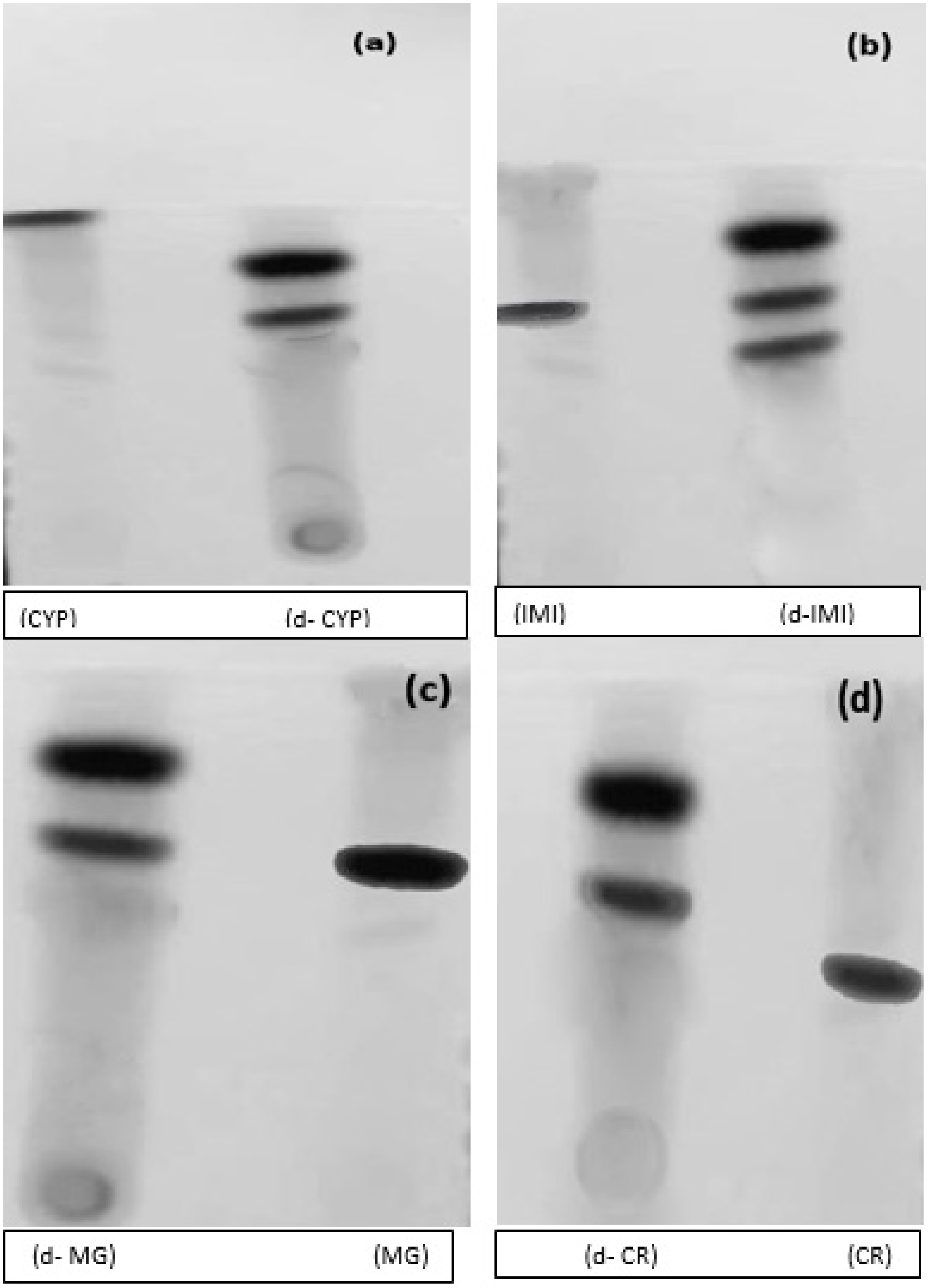

Figure S2. Thin Layer chromatography analysis of (a). CYP before and after biodegradation with P7, (b). IMI before and after degradation with P11, (c). MG before and after degradation with D6, (d). CR before and after degradation with D4.

d- CYP, d-IMI, d-MG, d-CR = degraded CYP, degraded IMI, degraded MG and degraded CR respectively. P7 = *B. thuringiensis* + *B. cereus*, P11 = *B. thuringiensis* + *E. hormaechei* + *Bacillus* sp. (5A) + *B. cereus*, D6 = *Bacillus* sp. (CF3) + *Bacillus* sp. (DF4) and D4 = *L. sphaericus* + *Bacillus* sp. (CF3).
